# Traditional mineral medicine realgar and *Realgar-Indigo naturalis formula* potentially exerted therapeutic effects by altering the gut microbiota

**DOI:** 10.3389/fmicb.2023.1143173

**Published:** 2023-04-18

**Authors:** Lu Luo, Chaofeng Li, Nanxi Huang, Qiaochu Wang, Zihao Zhang, Chen Song, Hui Yang, Maowen Yuan, Ziwen Xu, Jialei Sun, Zhijie Zhang

**Affiliations:** ^1^Institute of Chinese Materia Medica, China Academy of Chinese Medical Sciences, Beijing, China; ^2^Department of Biochemistry and Molecular & Cellular Biology, Georgetown University Medical Center, Washington, DC, United States; ^3^School of Pharmaceutical Sciences, Tsinghua University, Beijing, China; ^4^School of Earth Science and Resources, China University of Geosciences, Beijing, China; ^5^Third Affiliated Hospital of Zhejiang University of Traditional Chinese Medicine, Hangzhou, China

**Keywords:** realgar, *Realgar-Indigo naturalis formula* (RIF), gut microbiota, *Bacteroidales*, mineral medicine

## Abstract

**Introduction:**

Realgar has a long history ofuse in traditional medicines. However, the mechanism through which Realgar or *Realgar-Indigo naturalis formula* (RIF) exert therapeutic effects is only partially understood.

**Methods:**

In this study, 60 feces and 60 ileum samples from rats administered with realgar or RIF were collected to examine the gut microbiota.

**Results:**

The results showed that realgar and RIF influenced different microbiota in both feces and ileum. Compared with realgar, RIF at low dosage (0.1701 g/3 ml) significantly increased the microbiota diversity. LEfSe and random forest analyses showed that the bacterium *Bacteroidales* was significantly altered after RIF administration, and it was predicted that these microorganisms contribute to the inorganic arsenic metabolic process.

**Discussion:**

Our results suggest that realgar and RIF may exert their therapeutic effects through influencing microbiota. The low dose of RIF had greater effects on increasing the diversity of microbiota, and *Bacteroidales* in feces might participate in the inorganic arsenic metabolic process to exert therapeutic effects for realgar.

## Introduction

Minerals have been used within various traditional medicine systems since ancient times, and are still in use today (Luo et al., 2021c). They are found in many ethnopharmacy systems as herbal preparations including traditional Indian Ayurveda and Sri Lanka medicine (Kankanamalage et al., 2014; Joshi et al., 2017; Liu et al., 2018). Minerals in the forms of realgar are found in many traditional formulas, including Chinese medicines, where approximately 7% of the formulas contain cinnabar and/or realgar (Chinese Pharmacopoeia Commission, 2020). In the traditional Tamil system of Siddha Medicine, nearly half of the preparations used for diabetes treatments contain inorganics, including cinnabar and realgar (Sathasivampillai et al., 2017). Realgar is frequently included in traditional oral remedies for antipyretic, anti-inflammatory, antiulcer, anti-convulsive, and anti-schistosomiasis effects (Liu et al., 2011; Wu et al., 2011; Tian et al., 2014). Highly purified crystalline realgar is used for acute promyelocytic leukemia (APL) treatment with high efficacy and safety (Baláž et al., 2009; Mao et al., 2010).

Realgar is an arsenic sulfide mineral consisting of As_2_S_2_ and As_4_S_4_. Arsenic (As) is used mainly in the form of mineral arsenicals, including orpiment (As_2_S_3_), realgar (As_4_S_4_), and arsenolite (which contains arsenic trioxide, As_2_O_3_). It is structurally different from sodium arsenate (As^5+^) and sodium arsenite (As^3+^), and is the form used in oral traditional medicines (Liu et al., 2018). Arsenic sulfide As_4_S_4_ is a red semiconductor that exists in several crystalline forms. The room temperature stable α-As_4_S_4_ is structurally identical to mineral realgar (Mullen and Nowacki, 1972; Baláž et al., 2009). Due to the high lattice energy, realgar is poorly soluble in water and thus is considered to be less poisonous than arsenic trioxide (Wei et al., 2009). The sulfide forms of cinnabar and realgar are the basis of their therapeutic effects and safety after oral administration (Wang et al., 2008). It has been reported that the coarse realgar powder is only partially dissolved in the stomach and intestinal fluids with the total amounts of arsenic reaching 0.10 and 0.40%, respectively (Zhang et al., 2007). Inorganic arsenic was approved by Food and Drug Administration as a first line chemotherapeutic agent against certain hematopoietic cancers (Norsworthy et al., 2020). Realgar was found to be as effective as arsenic such as arsenic trioxide (As_2_O_3_, ATO), but with relatively good oral safety profiles even on chronic administration, which gives realgar an advantage over ATO in treatments. Orally administered realgar was shown in clinical trials as highly effective and safe for patients with APL (Wei et al., 2009). Although, reports propose that the sulfide forms of realgar is the basis of its therapeutic effects and safety, the mechanism of realgar in clinic use is currently not established (Wang et al., 2008).

*Realgar-Indigo naturalis formula* (RIF or Huang-Dai-Pian) was developed in the 1980s from a traditional medicine theory base, in which realgar is the principal element, and *Indigo naturalis [Baphicacanthus cusia (Nees) Bremek]*, *Salvia miltiorrhiza Bge*, and *Pseudostellaria heterophylla (Miq.) Pax* are adjuvant components to enhance the effects of realgar. The main active components of RIF are realgar, tetraarsenic tetrasulfide (A), indirubin (I), and tanshinone IIA (T). RIF can reduce the mortality of murine promyelocytic leukemia *in vivo* by intensifying ubiquitination/degradation of onco-protein, enhancing G (1)/G (0) arrest, and inducing AQP9 to facilitate the transportation of other ingredients into tumor cells. Clinical trials showed that RIF was effective for certain APL, with a remission rate of over 90% and a 5-year overall survival rate of 87% (Wang et al., 2008). RIF together with Imatinib Mesylate was effective against chromosome-positive chronic myeloid leukemia, with a recession rate of 42.75%, an improvement in effect than with Imatinib alone. Moreover, RIF was tolerable with moderate adverse effects, such as gastrointestinal discomfort and rash in patients suggesting that RIF is a better therapeutic intervention choice than realgar (Liu et al., 2018).

Although realgar and RIF are clinically effective, the main component of realgar, As_4_S_4_, is a water-insoluble mineral and cannot be absorbed by the human body. Therefore, it is still being determined how realgar and RIF work in the human body to achieve their therapeutic effects. *In vitro* simulation experiments showed that the intestine is the main absorption site of realgar, and gut microbiota converts realgar from an inorganic non-absorbable mineral to an organic absorbable ion and enables realgar to exert its therapeutic properties (Zhang et al., 2007). This suggests that microbiota may play a significant role in mediating the therapeutic effects of realgar and RIF. In this study, we designed a randomized animal experiment to explore whether realgar and RIF can exert their therapeutic effects by altering the microbiota structure of gut microbes. Rats were fed with realgar and RIF with different doses for eight consecutive days, feces and small intestines were collected for DNA extraction, and gut microbes were compared among different groups. This study aims to explore the appropriate dosage of realgar and RIF for safer and better clinical effect; the change of diversity of microbiota after realgar and RIF treatment; as well as the potential biomarker in microbiome for evaluation and underlying mechanism of therapeutic effect.

## Materials and methods

### Agent

The Traditional Chinese Medicine compound patent RIF (Huang Dai Pian) was provided by Anhui Tiankang Medical Technology Co., Ltd. The mineral medicine realgar was collected from Shimen, Hunan province in China. CMC-Na powder was provided by Shanghai McLean Biochemical Technology Co., Ltd.

### *In vitro* dissolution of soluble arsenic from realgar and RIF

The solution composition included artificial gastric fluid, artificial intestinal fluid, and 50% ethanol prepared according to the Chinese Pharmacopoeia Commission (2020) (see section “Methods” in Supplementary material). Three portions of artificial gastric juice, artificial intestinal juice, 50% absolute ethanol, or 16% hydrochloric acid were precisely mixed in a beaker with 500 ml each. Three portions of RIF tablets, 8 tablets each (about 0.27 g per tablet), were mixed with the solution. A total of 5 ml of samples were taken at 5, 10, 15, 20, 30, 45, 60, 90, and 120 min after the dissolution for future tests. A total of 5 ml of blank dissolution medium was added immediately after each extraction. For tests, samples were filtered through a 0.22 μm microporous membrane into a centrifuge tube. The realgar test solution was also prepared following the same method, and the realgar sample was 1 g each. A total of 0.3 ml of the sample solution was mixed with 0.3 ml of nitric acid for digestion. Then samples were diluted to 3 ml and shaken to obtain the test solution for detection by ICP-AES.

### Blood collection and blood arsenic test

A total of 180 specific pathogen-free (SPF) SD rats (half female and half male, 200 ± 20 g) were purchased from Beijing Weitong Lihua Laboratory Animal Technology Co., Ltd. After 3 days of acclimatization in a laboratory environment at 20°C and 45–65% relative humidity with a 12-h light/dark cycle, SPF SD rats were randomly divided into five groups [low dose *Realgar-Indigo Naturalis Formula* group (LRIF), high dose *Realgar-Indigo Naturalis Formula* group (HRIF), low dose Realgar group (LR), high dose Realgar group (HR), and control group (NC)]. Each group was further divided into six subgroups to protect the rats from excessive bleeding of blood sampling. For the single administration group, blood was collected after a single administration. For the first group, blood was collected at 0.5, 6, 24, and 64 h; for the second group, blood was collected at 1, 8, 30, and 72 h; for the third group, blood was collected at 1.5, 10, and 36 h; for the fourth group, blood was collected at 2, 12, and 42; for the fifth group, it was collected at 3, 16, and 48 h; for the sixth group, blood was collected at 4, 20, and 56 h. Approximately 0.7 ml of blood was collected from the carotid artery each time. A total of 5 ml blood was collected at the last time point (72 h) and divided into three groups, one 1 ml group, and two 2 ml groups. For the continuous administration group, rats were administrated for 8 consecutive days. The drug was administered at 9:00 a.m. every day, and 0.7 ml of blood was collected 3 h after administration. Rats were fed with food and water regularly during the period. Fasting for 8∼12 h before administration on the last day, blood was collected at 0.5, 1, 1.5, 2, 3, 4, 6, 8, 10, 12, 16, 20, 24, 30, 36, 42, 48, 56, 64, and 72 h, and about 0.7 ml of blood was collected from the carotid artery at each time point. At the last time point (72 h), 5 ml blood were collected and divide into three groups, one 1 ml group and two 2 ml groups. Then blood was transferred to a heparinized anticoagulation centrifuge tube and centrifuge immediately (10,000 r/min, 4°C, 10 min), then the upper plasma was collected and stored in a −20°C refrigerator for testing.

### Gut microbiota collection in rats

A total of 60 SPF SD rats (half female and half male, 200 ± 20 g) were purchased from Beijing Weitong Lihua Laboratory Animal Technology Co., Ltd. (Supplementary Table 1). After 3 days of acclimatization in a laboratory environment at 20°C and 45–65% relative humidity with a 12-h light/dark cycle, SPF SD rats were randomly divided into five groups. Rats in the LRIF group and HRIF group were orally administered with tablets dissolved in 5‰ CMC-Na. The rats in the low-dose realgar group and the high-dose realgar group (LR group and HR group) were given realgar dissolved in 5‰ CMC-Na. The control group (NC) was directly gavage with 5‰ CMC-Na solution. The specific doses of each group are as follows: LRIF (0.1701 g/3 ml), HRIF (0.3402 g/3 ml), LR (0.02722 g/3 ml), HR (0.05443 g/3 ml), control (0.015 g/3 ml), and all rats were fed for 8 days. The RIF dose is equivalent to an adult’s daily dose. The dose of the realgar group was calculated based on the RIF group (realgar accounted for 16% of the RIF tablet). At the end of day 8, all rats from the five groups were sacrificed, and their small intestines were removed. Feces samples were collected from rats for gut microbiota analysis before sacrifice. All samples were labeled and stored at −80°C.

### Bacterial DNA isolation and quantification

Metagenomic DNA for each sample was extracted using the QIAamp DNA Feces Mini Kit (Qiagen, Hamburg, Germany). The quality of metagenomic DNA was then evaluated by 1% gel electrophoresis, followed by specific amplification using a pair of universal primers (338F 5′-ACTCCTACGGGAGGCAGCA-3′, 806R 5′-GGACTACHVGGGTWTCTAAT-3′) targeting the V3–V4 hypervariable region of 16S rRNA gene. After PCR amplification, the quality of the products was accessed and purified using the AxyPrep DNA Gel Extraction Kit (Axygen Biosciences, Union City, CA, USA) according to the manufacturer’s instructions. Then, the purified amplicons were quantified by QuantiFluor-ST (Promega, Madison, WI, USA) and then sequenced under the MISEQ platform (Illumina, San Diego, CA, USA) of a commercial company (Majorbio Bio Pharm Technology Co., Ltd., Shanghai, China) (Krzywinski et al., 2009).

### Statistical and bioinformatics analyses

All raw sequencing data were deposited in the Sequence Read Archive (SRA) database of the National Center for Biotechnology Information (NCBI) with the accession number PRJNA690686 (bacteria). Operational taxonomic units (OTUs) were clustered using uparse software (version 7.1),^1^ with a similarity of 97%. Each 16S rRNA gene sequence was analyzed with the RDP classifier (version 2.2)^2^ based on the Silva 16S rRNA database with a confidence threshold of 70% (Oksanen and Simpson, 2023). Based on the OTUs, various alpha-diversity indexes [i.e., Chao estimate, observed species (Sobs), abundance-based coverage estimator (ACE), and Shannon and Simpson index] were used to evaluate the ecological features of the feces bacterial community in the sample. The alpha diversity index was calculated by the mothur software package (version V.1.30.1) using representative sequences of OTUs (≥97% similarity) and their relative abundances. Values were compared using the one-way ANOVA least significant difference (LSD) test, or the non-parametric Kruskal–Wallis test when the data were not normally distributed. In each case of the stated test, the *p*-value ≤ 0.05 was considered statistically significant. For group comparisons, the Bray–Curtis dissimilarity metric and permutational multivariate analysis of variance (PERMANOVA) were performed using the vegdist and adonis functions from the vegan package in the *R* programming environment (Berg et al., 2016). Taxonomic heat maps were clustered by the complete linkage method with the Bray–Curtis distance and generated by ggplot2. Circos plots showing the distribution of core microbiota were visualized using the Circos table viewer (Berg et al., 2016). Co-occurrence analyses were performed based on Spearman’s correlation scores among microbial taxa. Network visualization and property measurements were calculated with the interactive platform Gephi (Bastian et al., 2009). Linear discriminant analysis effect size (LEfSe) was applied to identify the biomarkers among different compartment niches (*p* < 0.05 and LDA score >4).

## Results

### Pharmacokinetics of realgar and RIF

**FIGURE 1:**
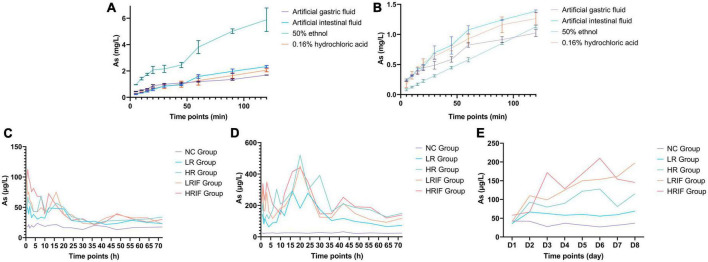
*In vitro* and *in vivo* dissolution of soluble arsenic from realgar and RIF. **(A)** The *in vitro* dissolution of realgar. **(B)** The *in vitro* dissolution of RIF. **(C)** The *in vivo* dissolution of realgar and RIF after single administrations of a low dose of RIF (LRIF), high dose of RIF (HRIF), low dose of realgar (LR), high dose of realgar (HR), or empty control (NC). **(D)** The *in vivo* dissolution of realgar and RIF after continuous administrations of LRIF, HRIF, LR, HR, or NC. **(E)** The *in vivo* dissolution of realgar and RIF during continuous administration of LRIF, HRIF, LR, HR, or NC. For *in vitro*, *N* = 3; for *In vivo*, *N* = 18.

The dissolution of realgar and RIF in different solvents including artificial gastric and intestinal fluid, was assessed. Realgar and RIF had higher dissolution in artificial intestinal fluid except for the dissolution of realgar in 50% ethanol suggesting that realgar and RIF were dissolved in the intestine (Figures 1A, B). The dissolution *in vivo* was further tested under different conditions. Rats were given single administration or continuous administration of a low dose of RIF (LRIF), high dose of RIF (HRIF), low dose of realgar (LR), high dose of realgar (HR), or empty control (NC), then As concentration in blood was measured. Continuous administration stabilized the concentration of blood As in rats compared with single administration (Figures 1C–E). Therefore, continuous administration of realgar and RIF was used for future animal studies. The concentration of As after continuous administration was also measured, and showed that the concentration of As decreased overtime (Figure 1D).

### Sequencing results

**FIGURE 2:**
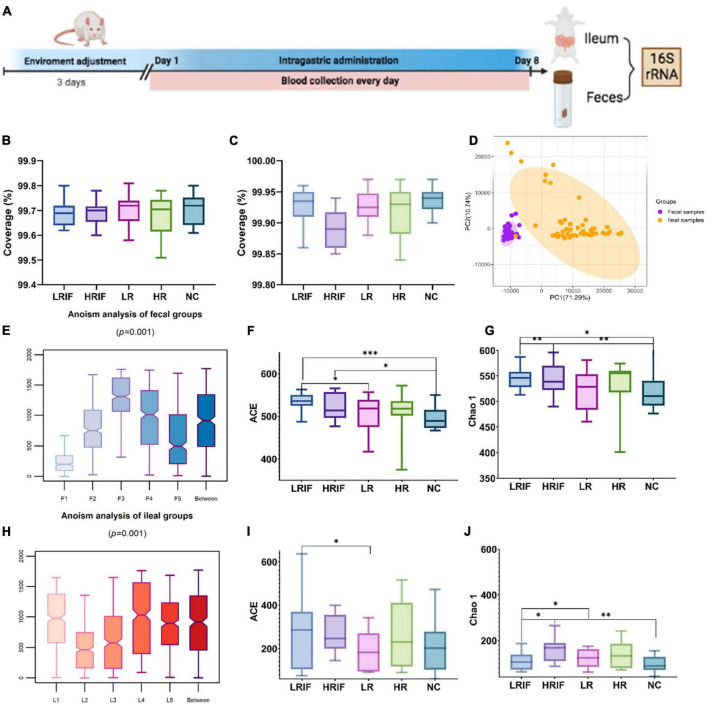
Phylogenetic diversity of gut microbiomes among LRIF, HRIF, LR, HR, and NC groups in fecal and ileal samples. **(A)** Graph explained the protocol of animal experiment. **(B)** Microbiota coverage of LRIF, HRIF, LR, HR, and NC groups in feces samples. **(C)** Microbiota coverage of LRIF, HRIF, LR, HR, and NC groups in ileum samples. **(D)** Principal component analysis (PCA) based on operational taxonomic unit (OTU) level microbiota composition of all feces and ileum samples (PC1 = 71.29%, PC2 = 10.74%). Feces samples are indicated by purple dots and ileum samples by orange dots. The colors denote individual samples. **(E)** Anosim analysis of gut microbiota based on unweighted UniFrac distance of feces samples. **(F)** ACE index of the microbiota from LRIF, HRIF, LR, HR, and NC groups in feces samples. **(G)** Chao1 index of the microbiota from LRIF, HRIF, LR, HR, and NC groups in feces samples. **(H)** Anosim analysis of gut microbiota based on unweighted UniFrac distance of ileum samples. **(I)** ACE index of the microbiota from LRIF, HRIF, LR, HR, and NC groups in ileum samples. **(J)** Chao1 index of the microbiota from LRIF, HRIF, LR, HR, and NC groups in ileum samples. For ACE and Chao1, the horizontal bars within boxes represent median. The tops and bottoms of boxes represent the 75th and 25th percentiles, respectively; *N* = 12. *P < 0.05, ^**^P < 0.01, ^***^P < 0.001.

Rats were administered with LRIF, HRIF, LR, HR, or NC for 8 days. A total of 60 feces (large intestine) samples and 60 ilea (small intestine) samples from 60 rats were collected (Figure 2A). We then generated bacterial profiles via targeting 16S ribosomal RNA gene using 338F and 806R primers followed by Illumina MiSeq sequencing (Supplementary Tables 2, 3).

### Gut microbiota alteration of feces and ileum after Realgar or RIF administrations

To understand the coverage of member microbiota in feces and ileum, Good’s coverage was calculated (Figures 2B, C), indicating that the majority of the bacterium types in the samples had been captured. We found that the composition of microbiota was different in feces and ileum. The principal component analysis (PCA) showed that the microbiota of feces and ileum formed two separate clusters, suggesting that realgar and RIF had different effects on feces and ileum samples (Figure 2D). We then explored the effects of different doses of realgar and RIF on the microbiota. PCA with Bray–Curtis dissimilarity showed that RIF and realgar group formed two data clusters in feces and ileum samples (Supplementary Figures 1, 2). Anosim analysis revealed a significant difference in microbiota among different doses of RIF and realgar groups with the *R* value of the unweighted in feces groups being *R* = 0.11 > 0 and *p* = 0.001 < 0.01, and that in ileum groups was *R* = 0.16 > 0 and *p* = 0.001 < 0.01. Thus, the grouping was reasonable (Figures 2E, H). Measurement of within-sample diversity (alpha-diversity) was used to evaluate the ecological features of the feces and ileum microbiota in the sample. Two measurements were used to avoid bias from a single measurement. All the feces samples showed increased intraindividual diversity compared to ileum samples, as indicated by the ACE and Chao 1 richness estimators. In feces samples, the LRIF group showed a significant intra-individual microbial richness compared with the control or HRIF group suggesting that a low dose of RIF recruited more microbiota (Figures 2F, G, I, J). These data suggest that a low dose of RIF has significantly increased the diversity of microbiota.

### Composition and variation in feces and ileum microbiota after realgar or RIF administrations

**FIGURE 3:**
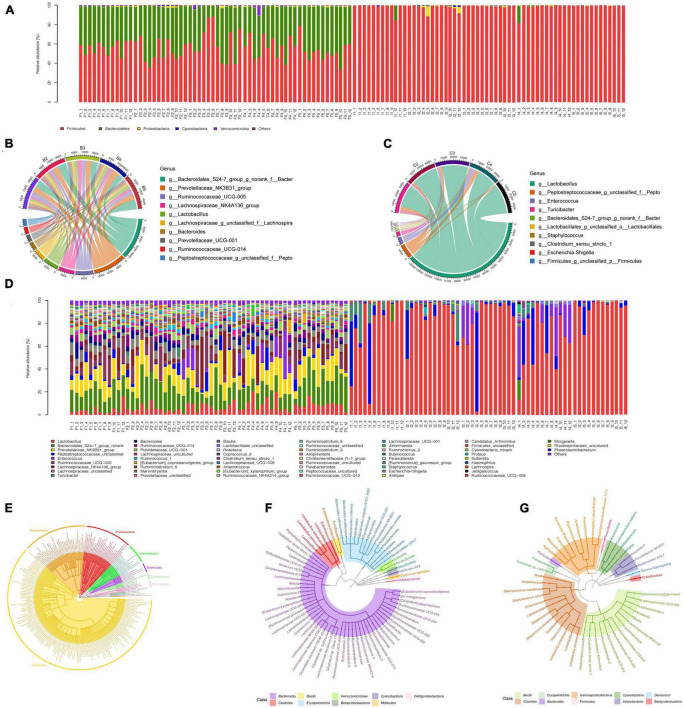
Gut microbiota composition analysis of feces and ileum samples. **(A)** Community bar plot analysis at the phylum level for all samples. F represents feces samples and I represents ileum samples. **(B)** Circos plot of the abundance of top ten dominant genus in feces. **(C)** Circos plot of the abundance of top ten dominant genus in ileum. **(D)** Community bar plot analysis at the genus level for all samples. F represents feces samples and I represents ileum samples. **(E)** Phylogenetic tree of the diversity of gut microbiota in both feces and ileum groups at the species level. **(F)** Phylogenetic tree of the diversity of gut microbiota in feces samples at the species level. **(G)** Phylogenetic tree of the diversity of gut microbiota in ileum samples at the species level.

We then explored the distribution of microbiota in each group. A phylogenetic tree was built with the method of Neighbor-Joining Tree using the representative sequences of the 73 OTUs in feces samples and 49 OTUs in ileum samples. We first examined the difference of microbiota between feces and ileum samples. In feces samples, OTU enriched belonged to two phyla. A total of 56.02% of feces samples were assigned into *Firmicutes* phylum, and 42.49% of feces samples were assigned to *Bacteroidetes*. In ileum samples, OTU enriched also belonged to the same two phyla. However, 98.46% of all the ileum samples were assigned into *Firmicutes* phylum, and only 0.66% *Bacteroidetes* was in ileum microbial population (Figure 3A). Consistent with results of phylum, feces samples had more evenly distributed microbiota such as *Prevotellaceae* (14.67%), *Bacteroidales* (9.17%), and *Ruminococcaceae* (7.27%) at the genus level. Ileum samples had limited distributed microbiota that mainly consisted of *Lactobacillaceae* (80.75%) and *Peptostreptococcaceae* (8.61%) at the genus level (Figures 3B–D). Feces samples had more evenly distributed microbiota such as *Prevotellaceae_NK3B31_group* (7.35%), *Prevotellaceae_NK3B32_group* (3.64%), *Prevotellaceae_ NK3B33_group* (3.34%) at the species level while *Lactobacillus intestinalis* (60.23%) dominated Ileum samples at the species level (Figures 3E–G). These results indicate a greater relative abundance of bacteria in feces compared with ileum.

### Interaction networks of the gut microbiota after realgar or RIF administrations

**FIGURE 4:**
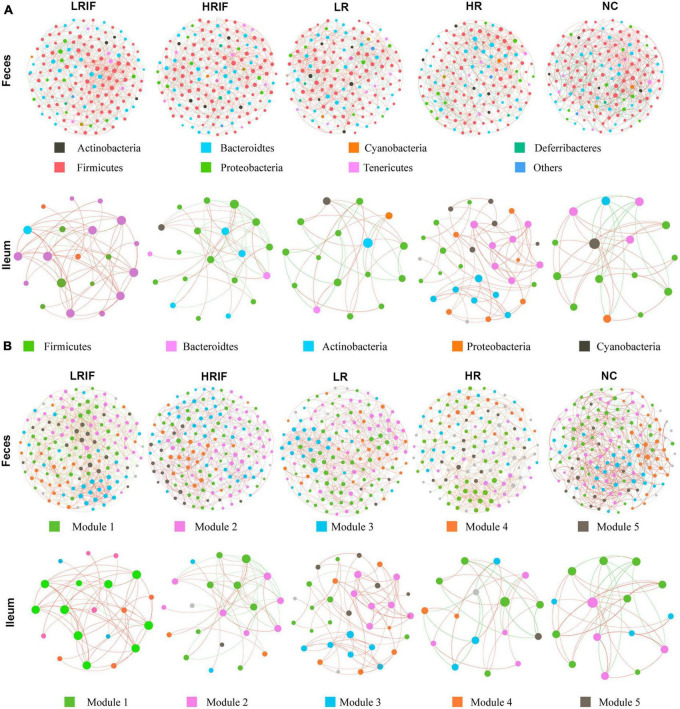
Co-occurrence network analyses of microbial communities in species and modular samples. **(A)** The OTUs in feces were grouped by color into seven phyla including *Actinobacteria, Bacteroidetes, Cyanobacteria, Deferribacteres, Firmicutes, Proteobacteria*, and *Tenericutes*; while OTUs in ileum were grouped by color into five phyla including *Firmicutes, Bacteroidetes, Actinobacteria, Proteobacteria*, and *Cyanobacteria*. **(B)** The OTUs in both feces and ileum were grouped into five major modules. Lines connecting two nodes represent a group of strong (*R* > 0.6) and significant correlations (*p* < 0.05). The red/orange and green/blue lines indicate positive and negative connections between the nodes. The size of each circle represents the relative abundance of each node.

To investigate the shifts of interaction networks with the gut microbiota in different realgar or RIF administration groups, we constructed co-occurrence networks of the gut microbiota with the OTUs that satisfied the following two criteria (Liu et al., 2019): (i) relative abundance was >0.05% and (ii) the OTUs were identified in more than 20% of the tested samples. The co-occurrence networks were constructed using the SparCC algorithm based on OTU abundances (Friedman and Alm, 2012). Clustering coefficients were calculated by the Molecular Complex Detection (MCODE) plugin in Cytoscape, and modularity was used to identify modules in the co-occurrence networks which showed the effects of realgar and RIF on microbial network complexity. In feces, the average degree of the LRIF group was ranked the highest (438.33), followed by HRIF (426.58), HR (415.42), LR (404.83), and NC (404). LRIF (0.523), also presented the highest degree of centrality, followed by HRIF (0.509), HR (0.496), LR (0.483), and NC (0.482). LRIF had the highest closeness centrality (0.531), followed by HRIF (0.524), HR (0.517), LR (0.511), and NC (0.509) (Figure 4A). The complexity of the phylogenetic co-occurrence networks was reflected in the average number of edges per node (Bader and Hogue, 2003). In feces, the complexity was the highest (14.75) in the LRIF group, and the complexity scores decreased from HRIF (13.42) to LR (12.83), HR (12.75), arriving at the lowest in the NC group (12.41). In ileum, the average number of edges per node of LRIF was ranked the highest (3.33), followed by NC (3.09), HRIF (2.75), HR (2.6), and LR (2.5) (Figure 4B). In feces, *Bacteroidetes* was identified as having the highest degree. Overall, we identified that the LRIF group had higher stability, complexity, and modality in the bacterial networks.

### Relative abundance of OTUs in feces and ileum microbiota after realgar or RIF administrations

**FIGURE 5:**
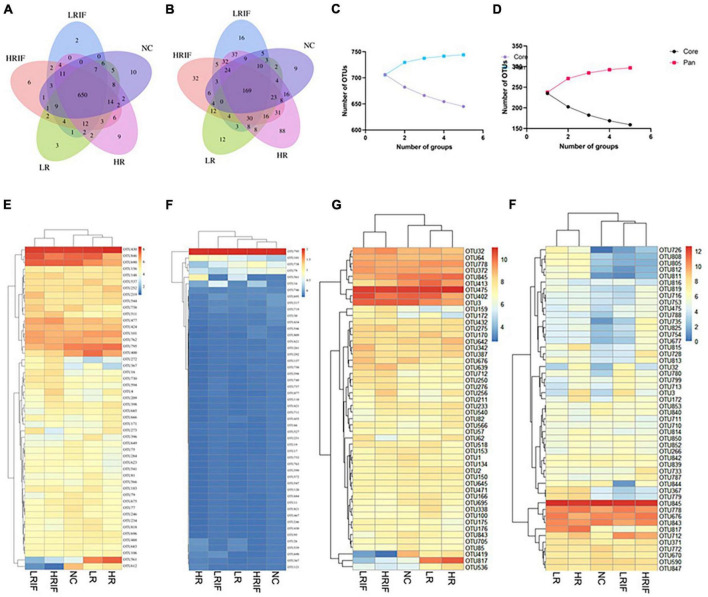
Relative abundance analyses at the OTU level of the microbial communities of the feces and ileum. **(A)** Venn diagram of shared and unique genera among five groups in fecal samples. **(B)** Venn diagram of shared and unique genera among five groups in ileal samples. **(C)** Pan and core OTUs of fecal samples. **(D)** Pan and core OTUs of ileal samples. **(E)** Heatmap showing the top 50 OTUs with most significant difference among five groups in fecal samples. **(F)** Heatmap showing the top 50 OTUs with most significant difference among five groups in ileal samples. **(G)** Heatmap showing the most abundant top 50 OTUs in fecal samples. **(H)** Heatmap showing the most abundant top 50 OTUs in ileal samples.

To further characterize the gut microbiota structure in realgar and RIF treatment groups, we analyzed the gut microbiota data at the OTU level (Supplementary Figure 5). Significant differences were observed among different realgar and RIF treatment groups. Among the top five abundant species in feces samples, realgar and NC groups shared several species including: *Rhodospirillaceae, Prevotella*, and *Barnesiella*, while RIF and NC group shared *Rhodospirillaceae, Sphingomonas*, and *Bacteroidales*, suggesting that realgar and RIF influenced different microbiota (Figures 5A, B). The unique abundant species shared between RIF and NC group is *Bacteroidales*. As per fecal microbiota, *Bacteroidales_S24-7_group* and *Bacteroides_vulgatus_ATCC_8482* presented more abundance in LRIF and HRIF groups. Furthermore, PERMANOVA analysis showed significant microbial community differences of *Bacteroidales_S24-7_group* in feces (Figures 5C–E). These data suggest that *Bacteroidales* could be a biomarker to evaluate the effects of RIF on the diversity of microbiota.

Among the top five abundant species in ileum, realgar and NC groups share three species including *Prevotella, Pseudomonas*, and *Defluviitaleaceae* while RIF did not share species with the NC group suggesting that RIF influences microbiota in a manner different from realgar. *Christensenellaceae*, *Lachnospiraceae*, *Bacteroides acidifaciens*, *Marvinbryantia*, and *Parabacteroides* are uniquely enriched in the LRIF group. PERMANOVA analysis showed significant microbial community differences in the feces of *Lactobacillales* (Figures 5D–F). The influence of RIF on microbiota in ileum requires further investigation.

### Microbiota biomarker in feces and ileum after realgar and RIF administrations

**FIGURE 6:**
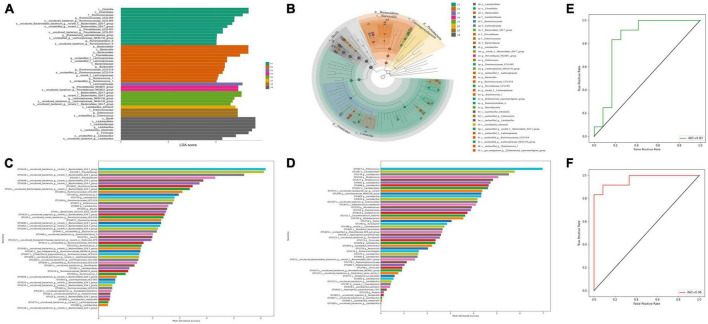
Linear regression analysis between bacterial community structure and function. **(A)** Linear discriminant effect size (LEfSe) analysis of microbial communities in feces and ileum samples with linear discriminant analysis (LDA) scores indicating significant differences in the microbiota among separate groups. (F1 represents LRIF group, F2 represents HRIF, F3 represents LR, F4 represents HR, and F5 represents control in feces; L2 represents HRIF, L4 represents HR, and L5 represents control in ileum). **(B)** Cladogram indicating the phylogenetic distribution of microbiota correlated with the feces and ileum groups. **(C)** Top 50 important biomarkers selected by random forest analysis in feces. **(D)** Top 50 important biomarkers selected by random forest analysis in ileum. The horizontal axis is the measurement standard of variables of importance, and the value is equal to the measurement value of variables of importance/standard deviation. The vertical axis is the variable names sorted by importance. **(E)** ROC curve (AUC) of gut-microbiota-based classification. Random forest classifiers were used to separate five groups in feces based on the OTU-level gut microbiome composition. **(F)** ROC curve (AUC) of gut-microbiota-based classification based on the random forest to separate five groups in ileum. The AUC value is the area under the corresponding curve. The diagnostic effect is better when the AUC > 0.5 and the AUC value is closer to 1.

We then tried to identify whether *Bacteroidales* can be used as a biomarker to evaluate the effects of RIF on the diversity of microbiota. We performed linear discriminant analysis coupled with effect size (LEfSe) on the taxa that exhibited linear discriminant analysis (LDA) scores greater than four. As we expect, phyla *Bacteroidetes* and classes *Bacteroidia* among OTUs were among represented OTUs (Figures 6A, B). Again, the statistically significant abundances were more enriched in the feces microbial community and LRIF presented a majority of the distinguishable OTUs. To determine whether OTUs could serve as biomarkers and which OTUs play important roles, we constructed a random forest model. The mean decrease in accuracy of the top 50 important variables is shown in Figures 6C, D. *Bacteroidales_S24-7_group* (OUT 419) in feces and *Enterococcus* (OTU 817) in ileum were annotated to the family of *Bacteroidales* and the genus of *Enterococcus*, respectively. The area under the ROC curve (AUG) was 0.83 and 0.96, respectively (Figures 6E, F). These data suggest that among biomarkers identified in this study, *Bacteroidales* is one important biomarker of RIF.

### The functional profiles of microbiota in feces and ileum after realgar or RIF administrations

**FIGURE 7:**
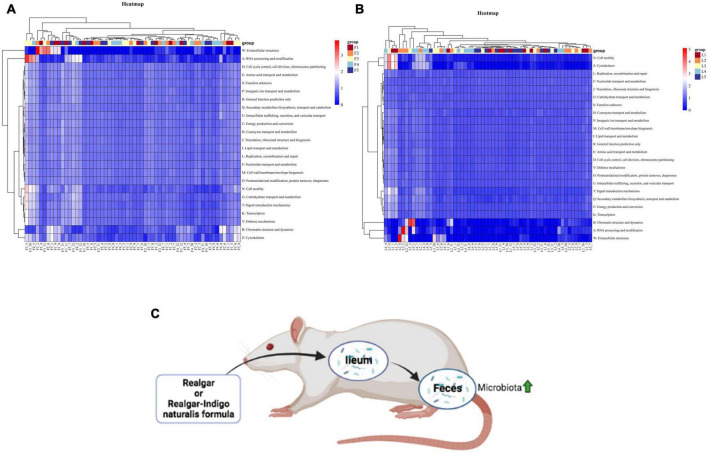
Heatmap of predicted functions based on COG analysis with PICRUSt. **(A)** Bacterial functional categories annotated by PICRUSt in feces. **(B)** Bacterial functional categories annotated by PICRUSt in ileum. [There are 95% probabilities of predicting the functions of gut microbiota (*p* < 0.05).] **(C)** The summarization of realgar and RIF effects on microbiota.

We used the OTUs in each group to predict functional profiles based on COG databases by PICRUSt (Phylogenetic Investigation of Communities by Reconstruction of Unobserved States). A total of 24 functional categories and 4,792 functional descriptions were obtained in feces and ileum microbial communities. The relative abundances of functional profiles with bacterial taxa in each group were presented in Figure 7. Metabolism functions including coenzyme transport and metabolism, amino acid transport and metabolism, nucleotide transport and metabolism, and lipid transport and metabolism were the main functions performed by bacteria in all five groups in feces. Especially, many OTUs were assigned to inorganic ion transport and metabolism, suggesting that microbiota metabolized As from realgar or RIF (Supplementary Figures 3, 4).

## Discussion

Although realgar and RIF have been approved to be clinically effective, the main component of realgar and RIF is a water-insoluble mineral (As_4_S_4_). How realgar and RIF work in the human body to achieve their therapeutic effects is unknown. To the authors’ knowledge this is the first time the effects of realgar and RIF on gut microbiota have been studied and elucidated. This study showed that realgar and RIF can influence different microbiota. RIF at a low dosage significantly increased the diversity of microbiota compared with RIF at a high dose and realgar at a low or high dose. LEfSe and random forest analyses showed that the *Bacteroidales* in feces was important after RIF administration (Figure 7C). Taken together, these data suggest that RIF at low dose should have optimal therapeutic effect and *Bacteroidales* in feces is a biomarker to evaluate the effects of RIF on the diversity of microbiota.

### Gut microbiota played a significant role in arsenic metabolism *in vivo*

Metals such as As play distinct roles in human anatomy. For example, recent studies identified some metals as “metalloestrogens” that activate estrogen receptor α through an interaction with its ligand binding domain and mimic estradiol to activate estrogen receptor α (Divekar et al., 2011; Cyrus et al., 2021). Therefore, it is essential to study how metals enter and metabolized by the human body. After the realgar enters the human body, a minor proportion of realgar will be absorbed by the body and methylated to form organic compounds and proteins (Xu et al., 2013). Inorganic arsenic from realgar and glutathione will form arsenic triglutathione and bind to macromolecular proteins to form As-containing proteins such as AsB and AsC (Janasik et al., 2015). During this process, gut microbiota also participates in arsenic metabolism. Previous studies have shown that gut microbiota affected the transformation and bioavailability of orally ingested soil As in the human gastrointestinal tract (Yin et al., 2022). The study also showed that As (III) was adsorbed through microbial reduction to increase bio accessibility of As (Yin et al., 2015). In this study, the biodiversity of microbiota was increased with realgar and RIF administration suggesting that realgar and RIF influenced the structure of gut microbiota, which, in turn, may contribute to the metabolism of As from realgar and RIF. These data suggest that realgar and RIF may exert therapeutic effects by influencing the metabolism of As of gut microbiota.

### Feces showed more diversified microbiota than ileum

Apparently, fecal samples showed a much more diversified microbiota regarding the richness and evenness, OUT numbers, as well as the modularity, complexity and connectivity of bacterial networks. In general, RIF groups showed higher richness and diversity than realgar groups, as well as the treatment groups over the control group. Most importantly, the intra-individual microbial richness, the stability, degree centrality, and the average degree of the bacterial co-occurrence networks ranks the highest in the LRIF group comparing to all other groups in both feces and ileum. These results indicated that the arsenic did impact the gut microbiota and altered its original bacterial structure of rats and RIF at a lower dosage presented a better result.

*Firmicutes* (56.02%) and *Bacteroidetes* (42.49%) occupied the majority of the phyla population in feces, while *Firmicutes* (98.46%) was the most predominant in ileum. *Clostridia* (22.68%) and *Bacteroidia* (21.29%) constituted the most prevalent class phenotype in feces, however, in it was *Bacilli* (86.04%) in ileum. *Clostridiales* (22.68%) and *Bacteroidales* (21.29%) dominated the feces orders while *Lactobacillales* (85.76%) was the most abundant order in ileum. *Ruminococcaceae* (10.38%), *Lachnospiraceae* (10.28%), and *Prevotellaceae* (9.76%), and *Bacteroidales* (9.51%) were most prevalent families in feces, and *Lactobacillaceae* (80.75%), *Peptostreptococcaceae* (8.61%), and *Enterococcaceae* (4.47%) were the most abundant in ileum. *Prevotellaceae* (14.67%), *Bacteroidales* (9.17%), and *Ruminococcaceae* (7.27%) are the most dominant genus in feces. While *Lactobacillus* (80.75%), *Peptostreptococcaceae* (8.61%), and *Enterococcus* (4.47%) occupied the majority of the ileal genus. *Bacteroidales* and *Ruminococcaceae* were increased in LRIF and LR groups comparing to HRIF and HR groups, while *Lachnospiraceae* and *Enterococcus* were decreased dramatically in LRIF and LR groups. *Enterococcus* (OTU817, 7735) has the highest weight among all OTUs in feces, while 26 *Bacteroidetes*, 24 *Firmicutes*, and 2 *Proteobacteria* in feces showed the highest degree.

### Lower dosage of *Realgar-Indigo naturalis formula* exhibited the best effective result

Arsenic accumulates in tissues especially in the heart, liver, spleen, lung, kidney, uterus and ovary, and the distribution and elimination of arsenic in the blood is slow. Drug metabolism studies showed that the renal excretion of realgar only accounts for 0.0367% of the dose, and the total arsenic excretion in feces accounts for 97.7% of the dose, indicating that realgar is mainly excreted through the gastrointestinal tract (Koch et al., 2007). Similarly, Chi et al. (2020) found that low bio-accessibility of arsenic (<40%) was observed in the digestive tract while bio-accessibility of arsenic was significantly (22%) higher in the active colon phase. Interestingly, study of this showed that exposure to As (III) in mice had structural and functional influence on the large intestine but had slight influence on the small intestine (Chiocchetti et al., 2019). This study examined both feces and ileum samples. Consistent with previous results, our results suggest that RIF has significantly increased the diversity of feces microbiota. Our results also showed that feces samples (large intestine) had more diversified microbiota while ileum samples (small intestine) had less diversified microbiota suggesting that microbiota in the large intestine mediates the effects of RIF. Also, high concentrations of metals are toxic (Luo et al., 2021a,b). To avoid toxicity of arsenic, it is essential to control the dose of RIF that contain arsenic in clinical use. As expected, the low dose of RIF increased the diversity of gut microbiota in feces microbiota in relation to the richness and evenness, OUT numbers, as well as the modularity, complexity and connectivity of bacterial networks; these together suggest that RIF at low dose may have better therapeutic effects.

### Biomarkers *Bacteroidales* in feces indicated the shift of gut microbiota after RIF administration

*Bacteroidales* is important. For example, *Bacteroides* belongs to *Bacteroidales* Gram-negative bacteria and is among the most abundant anaerobes in the human colon (Wexler, 2007). Many human *Bacteroides* species pose a substantial number of carbohydrate utilization genes and break down carbohydrates in the gut, such as dietary fiber and resistant starch, which the body can digest. *Bacteroides* also plays a vital role in the metabolism of protein, carcinogens, and xenobiotics (Veeranagouda et al., 2019). *Bacteroides* hosts molecular interaction to immune system development, provides protection from pathogens, and supplies nutrients to other microbial residents of the gut (Degnan and Macfarlane, 1995; Cameron et al., 2012; Sears et al., 2014; Collins and Patterson, 2020). Interestingly, other studies showed that As (III) resulted in enhanced numbers of the *Bacteroidetes* (Ignacio et al., 2015). These observations are consistent with our results, showing that *Bacteroidales* is a biomarker to evaluate the effects of RIF on the diversity of microbiota. These results indicate that *Bacteroidales* responds to As in a manner different from other bacteria. Intestine *Bacteroidetes* is also identified to be metal resistant. Bacteria can transport metal internally through non-specific metal homeostasis or specific membrane transport systems. Metals are then detoxicated by enzyme or cation diffusion facilitator systems and transported externally by efflux system. Moreover, *Bacteroides vulgatus* contains genes czcA, czcB, and czcC, which are essential for metal resistance (Sun et al., 2020). Interestingly, *B. vulgatus* is abundant in LRIF and HRIF groups, suggesting that it may be important in the therapeutic effects of RIF. Therefore, it is essential to investigate the mechanism of As metabolism by *Bacteroides* and *B. vulgatus* in the future.

## Conclusion

This study showed that realgar and RIF changed the gut microbiota structure in rats’ feces and ileum. RIF at a lower dosage had better effects on microbiota in feces, and *Bacteroidales* was important for evaluating the diversity of microbiota after RIF administration and possibly participated in the inorganic arsenic metabolic process to exert therapeutic effects for realgar. Therefore, we suggest the following in the clinical application of realgar and RIF: 1. RIF should be used to replace realgar to achieve better clinic results; 2. When RIF is used clinically, the dosage should be controlled to the levels shown in this research to achieve optimal clinical results.

## Data availability statement

The datasets presented in this study can be found in online repositories. The names of the repository/repositories and accession number(s) can be found in the article/Supplementary material.

## Ethics statement

The animal study was reviewed and approved by the China Academy of Chinese Medical Sciences.

## Author contributions

LL: investigation, writing—original draft, writing—review and editing, formal analysis, funding acquisition, and supervision. CL: investigation, formal analysis, and writing—review and editing. NH and QW: formal analysis, writing—original draft, and writing—review and editing. ZhZ, CS, JS, and HY: formal analysis. MY and ZX: data analysis and organization. ZjZ: writing—review and editing, funding acquisition, supervision. All authors contributed to the article and approved the submitted version.

## Footnotes

1
http://drive5.com/uparse/

2
http://sourceforge.net/projects/rdp-classifier/

## References

[B1] Bader G. D. Hogue C. W. V. (2003). An automated method for finding molecular complexes in large protein interaction networks. *BMC Bioinformatics* 4:1–27. 10.1186/1471-2105-4-2/FIGURES/12 12525261 PMC149346

[B2] Baláž P. Fabián M. Pastorek M. Cholujová D. Sedlák J. (2009). Mechanochemical preparation and anticancer effect of realgar As4S4 nanoparticles. *Mater. Lett.* 63 1542–1544. 10.1016/J.MATLET.2009.04.008

[B3] Bastian M. Heymann S. Jacomy M. (2009). Gephi: An open-source software for exploring and manipulating networks. *Proc. Int. AAAI Conf. Web Soc. Med.* 3 361–362. 10.1609/ICWSM.V3I1.13937

[B4] Berg M. Stenuit B. Ho J. Wang A. Parke C. Knight M. (2016). Assembly of the *Caenorhabditis elegans* gut microbiota from diverse soil microbial environments. *ISME J.* 10 1998–2009. 10.1038/ismej.2015.253 26800234 PMC5029150

[B5] Cameron E. A. Maynard M. A. Smith C. J. Smith T. J. Koropatkin N. M. Martens E. C. (2012). Multidomain carbohydrate-binding proteins involved in *Bacteroides thetaiotaomicron* starch metabolism. *J. Biol. Chem.* 287 34614–34625. 10.1074/jbc.M112.397380 22910908 PMC3464567

[B6] Chi H. Hou Y. Li G. Zhang Y. Coulon F. Cai C. (2020). In vitro model insights into the role of human gut microbiota on arsenic bio accessibility and its speciation in soils. *Environ. Pollut.* 263:114580. 10.1016/J.ENVPOL.2020.114580 33618458

[B7] Chinese Pharmacopoeia Commission (2020). *Pharmacopoeia of the People’s Republic of China.* Beijing: China Medical Science Press.

[B8] Chiocchetti G. M. Domene A. Kühl A. A. Zúñiga M. Vélez D. Devesa V. (2019). In vivo evaluation of the effect of arsenite on the intestinal epithelium and associated microbiota in mice. *Arch. Toxicol.* 93 2127–2139. 10.1007/S00204-019-02510-W/FIGURES/7 31309260

[B9] Collins S. L. Patterson A. D. (2020). The gut microbiome: An orchestrator of xenobiotic metabolism. *Acta Pharm. Sin. B* 10 19–32. 10.1016/J.APSB.2019.12.001 31998605 PMC6984741

[B10] Cyrus K. Wang Q. Sharawi Z. Noguchi G. Kaushal M. Chang T. (2021). Role of calcium in hormone-independent and -resistant breast cancer. *Int. J. Cancer* 149 1817–1827. 10.1002/IJC.33745 34289100 PMC9682976

[B11] Degnan B. A. Macfarlane G. T. (1995). Carbohydrate utilization patterns and substrate preferences in *Bacteroides thetaiotaomicron*. *Anaerobe* 1 25–33. 10.1016/S1075-9964(95)80392-0 16887504

[B12] Divekar S. D. Storchan G. B. Sperle K. Veselik D. J. Johnson E. Dakshanamurthy S. (2011). The role of calcium in the activation of estrogen receptor-alpha. *Cancer Res.* 71 1658–1668. 10.1158/0008-5472.CAN-10-1899/649463/AM/THE-ROLE-OF-CALCIUM-IN-THE-ACTIVATION-OF-ESTROGEN 21212417 PMC3057389

[B13] Friedman J. Alm E. J. (2012). Inferring correlation networks from genomic survey data. *PLoS Comput. Biol.* 8:e1002687. 10.1371/JOURNAL.PCBI.1002687 23028285 PMC3447976

[B14] Ignacio A. Nakano V. AvilaCampos M. (2015). Intestinal *Bacteroides vulgatus* showing resistance to metals. *Appl. Med. Res.* 1:46. 10.5455/amr.20150401012721

[B15] Janasik B. Reszka E. Stanislawska M. Wieczorek E. Fendler W. Wasowicz W. (2015). Biological monitoring and the influence of genetic polymorphism of As3MT and GSTs on distribution of urinary arsenic species in occupational exposure workers. *Int. Arch. Occup. Environ. Health* 88 807–818. 10.1007/S00420-014-1009-7/TABLES/6 25491248 PMC4508369

[B16] Joshi V. K. Joshi A. Dhiman K. S. (2017). The ayurvedic pharmacopoeia of India, development and perspectives. *J. Ethnopharmacol.* 197 32–38. 10.1016/J.JEP.2016.07.030 27404231

[B17] Kankanamalage T. N. M. Dharmadasa R. M. Abeysinghe D. C. Wijesekara R. G. S. (2014). A survey on medicinal materials used in traditional systems of medicine in Sri Lanka. *J. Ethnopharmacol.* 155 679–691. 10.1016/J.JEP.2014.06.016 24933220

[B18] Koch I. Sylvester S. Lai V. W. M. Owen A. Reimer K. J. Cullen W. R. (2007). Bioaccessibility and excretion of arsenic in Niu Huang Jie Du Pian pills. *Toxicol. Appl. Pharmacol.* 222 357–364. 10.1016/J.TAAP.2006.12.005 17239412

[B19] Krzywinski M. Schein J. Birol I. Connors J. Gascoyne R. Horsman D. (2009). Circos: An information aesthetic for comparative genomics. *Genome Res.* 19 1639–1645. 10.1101/GR.092759.109 19541911 PMC2752132

[B20] Liu H. Chen X. Hu X. Niu H. Tian R. Wang H. (2019). Alterations in the gut microbiome and metabolism with coronary artery disease severity. *Microbiome* 7 1–14. 10.1186/S40168-019-0683-9/FIGURES/4 31027508 PMC6486680

[B21] Liu J. Liang S. X. Lu Y. F. Miao J. W. Wu Q. Shi J. S. (2011). Realgar and realgar-containing Liu-Shen-Wan are less acutely toxic than arsenite and arsenate. *J. Ethnopharmacol.* 134 26–31. 10.1016/J.JEP.2010.11.052 21129479

[B22] Liu J. Wei L. X. Wang Q. Lu Y. F. Zhang F. Shi J. Z. (2018). A review of cinnabar (HgS) and/or realgar (As4S4)-containing traditional medicines. *J. Ethnopharmacol.* 210 340–350. 10.1016/J.JEP.2017.08.037 28864167

[B23] Luo L. Yang J. Wang C. Wu J. Li Y. Zhang X. (2021c). Natural products for infectious microbes and diseases: An overview of sources, compounds, and chemical diversities. *Sci. China Life Sci.* 65 1123–1145. 10.1007/S11427-020-1959-5 34705221 PMC8548270

[B24] Luo L. Dong L. Huang Q. Ma S. Fantke P. Li J. (2021a). Detection and risk assessments of multi-pesticides in 1771 cultivated herbal medicines by LC/MS-MS and GC/MS-MS. *Chemosphere* 262:127477. 10.1016/J.CHEMOSPHERE.2020.127477 32799136

[B25] Luo L. Wang B. Jiang J. Fitzgerald M. Huang Q. Yu Z. (2021b). Heavy metal contaminations in herbal medicines: Determination, comprehensive risk assessments, and solutions. *Front. Pharmacol.* 11:2016. 10.3389/FPHAR.2020.595335/BIBTEX PMC7883644 33597875

[B26] Mao J.-H. Sun X.-Y. Liu J.-X. Zhang Q.-Y. Liu P. Huang Q.-H. (2010). As4S4targets RING-type E3 ligase c-CBL to induce degradation of BCR-ABL in chronic myelogenous leukemia. *Proc. Natl. Acad. Sci.* 107 21683–21688. 10.1073/pnas.1016311108 21118980 PMC3003020

[B27] Mullen D. J. E. Nowacki W. (1972). Refinement of the crystal structures of realgar, AsS and orpiment, As2S3. *Zeitschrift für Kristallographie - Crystalline Materials* 136, 48–65. 10.1524/zkri.1972.136.16.48

[B28] Norsworthy K. J. Avagyan A. Bird S. T. Li Y. Akhtar S. Liao J. (2020). Second cancers in adults with acute promyelocytic leukemia treated with or without arsenic trioxide: A SEER-medicare analysis. *Leukemia* 34 3082–3084. 10.1038/s41375-020-0905-y 32518418

[B29] Oksanen J. Simpson G. L. (2023). *The vegan Package BiodiversityR View project ICRAF genetic resources unit View project.* Available online at http://cran.r-project.org/ (accessed November 21, 2022).

[B30] Sathasivampillai S. v Rajamanoharan P. R. S. Munday M. Heinrich M. (2017). Plants used to treat diabetes in Sri Lankan Siddha Medicine – An ethnopharmacological review of historical and modern sources. *J. Ethnopharmacol.* 198 531–599. 10.1016/J.JEP.2016.07.053 27448453

[B31] Sears C. L. Geis A. L. Housseau F. (2014). *Bacteroides* fragilis subverts mucosal biology: From symbiont to colon carcinogenesis. *J. Clin. Invest.* 124 4166–4172. 10.1172/JCI72334 25105360 PMC4191034

[B32] Sun Y. T. Xu H. H. Nie Y. Wang Y. G. Ma Z. C. Zhou W. (2020). Preliminary study of Realgar and arsenic trioxide on gut microbiota of mice. *Zhongguo Zhong Yao Za Zhi* 45 142–148. 10.19540/J.CNKI.CJCMM.20190813.403 32237423

[B33] Tian Y. Wang X. Xi R. Pan W. Jiang S. Li Z. (2014). Enhanced antitumor activity of realgar mediated by milling it to nanosize. *Int. J. Nanomed.* 9:745. 10.2147/IJN.S56391 24516332 PMC3916444

[B42] Veeranagouda Y. Husain F. Wexler H. M. (2019). Transposon mutagenesis of *Bacteroides fragilis*. *Microb. Trans. Mutag. Protoc. Appl*. 1137, 105–116. 10.1007/978-1-4939-9570-7_10 31197713

[B34] Wang L. Zhou G. B. Liu P. Song J. H. Liang Y. Yan X. J. (2008). Dissection of mechanisms of Chinese medicinal formula Realgar-Indigo naturalis as an effective treatment for promyelocytic leukemia. *Proc. Natl. Acad. Sci. U.S.A.* 105 4826–4831. 10.1073/PNAS.0712365105/SUPPL_FILE/12365FIG9.PDF 18344322 PMC2290784

[B35] Wei L. Liao P. Wu H. Li X. Pei F. Li W. (2009). Metabolic profiling studies on the toxicological effects of realgar in rats by 1H NMR spectroscopy. *Toxicol. Appl. Pharmacol.* 234 314–325. 10.1016/J.TAAP.2008.11.010 19073202

[B36] Wexler H. M. (2007). *Bacteroides*: The good, the bad, and the nitty-gritty. *Clin. Microbiol. Rev.* 20 593–621. 10.1128/CMR.00008-07 17934076 PMC2176045

[B37] Wu J. Shao Y. Liu J. Chen G. Ho P. C. (2011). The medicinal use of realgar (As4S4) and its recent development as an anticancer agent. *J. Ethnopharmacol.* 135 595–602. 10.1016/J.JEP.2011.03.071 21497649

[B38] Xu W. Wang H. Chen G. Li W. Xiang R. Pei Y. (2013). 1H NMR-based metabonomics study on the toxicity alleviation effect of other traditional Chinese medicines in Niuhuang Jiedu tablet to realgar (As2S2). *J. Ethnopharmacol.* 148 88–98. 10.1016/J.JEP.2013.03.073 23583735

[B39] Yin N. Cai X. Wang P. Feng R. Du H. Fu Y. (2022). Predictive capabilities of in vitro colon bioaccessibility for estimating in vivo relative bioavailability of arsenic from contaminated soils: Arsenic speciation and gut microbiota considerations. *Sci. Total Environ.* 818:151804. 10.1016/J.SCITOTENV.2021.151804 34808186

[B40] Yin N. Zhang Z. Cai X. Du H. Sun G. Cui Y. (2015). In vitro method to assess soil arsenic metabolism by human gut microbiota: Arsenic speciation and distribution. *Environ. Sci. Technol.* 49 10675–10681. 10.1021/ACS.EST.5B03046/ASSET/IMAGES/LARGE/ES-2015-030466_0003.JPEG 26248026

[B41] Zhang J. Zhang X. Ni Y. Yang X. Li H. (2007). Bioleaching of arsenic from medicinal realgar by pure and mixed cultures. *Process Biochem.* 42 1265–1271. 10.1016/J.PROCBIO.2007.05.021

