# Supplementary material for: Traditional mineral medicine realgar and Realgar-Indigo naturalis formula potentially exerted therapeutic effects by altering the gut microbiota
Source: Front Microbiol. 2023 Apr 18;14:1143173. doi: 10.3389/fmicb.2023.1143173 (PMC10151705; doi:10.3389/fmicb.2023.1143173)
Supplement: Supplementary file 1 [file Data_Sheet_1.docx]

**Supplementary Methods**

***In vitro* dissolution of soluble arsenic from realgar and RIF**

The configuration of each dissolution medium was made according to the 2020 edition of the Chinese Pharmacopoeia. Artificial gastric juice: take 16.4 ml of dilute hydrochloric acid, add about 800 ml of water and 10 g of pepsin, shake well, and dilute it to 1000 ml with water. Artificial intestinal juice: phosphate buffered saline (containing pancreatin) (pH=6.8). Take 6.8 g of potassium dihydrogen phosphate, add 500 ml of water to dissolve, adjust the pH value to 6.8 with 0.1mol/L sodium hydroxide solution; take another 10g of pancreatin, add an appropriate amount of water to dissolve, after mixing the two liquids, add water to dilute it to 1000 ml. 50% ethanol: take 500 ml of absolute ethanol and add deionized water to 1000 ml. 0.16% hydrochloric acid: the concentration of hydrochloric acid in the laboratory is 36%, so take 2000 ml of deionized water and add 8.89 ml of hydrochloric acid.
