# Supplementary material for: Traditional mineral medicine realgar and Realgar-Indigo naturalis formula potentially exerted therapeutic effects by altering the gut microbiota
Source: Front Microbiol. 2023 Apr 18;14:1143173. doi: 10.3389/fmicb.2023.1143173 (PMC10151705; doi:10.3389/fmicb.2023.1143173)
Supplement: Supplementary file 2 [file Data_Sheet_2.docx]

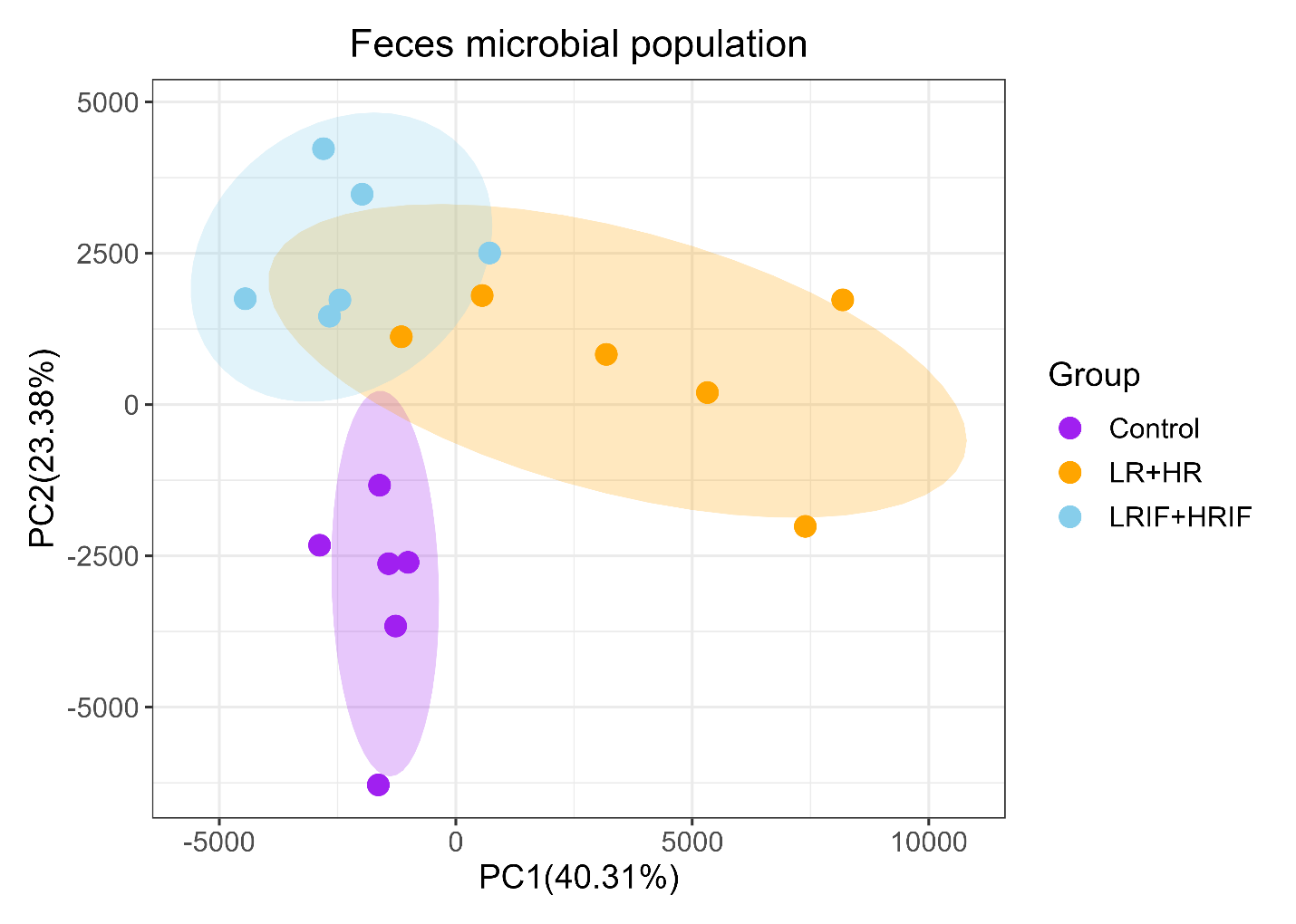


**Supplement Figure 1.** Beta-diversity of representative bacterial was assessed through conducting the PCA based on Bray-Curtis dissimilarity. The representative samples from microbial community among the LR+HR, LRIF+HRIF, and control groups were different in feces.


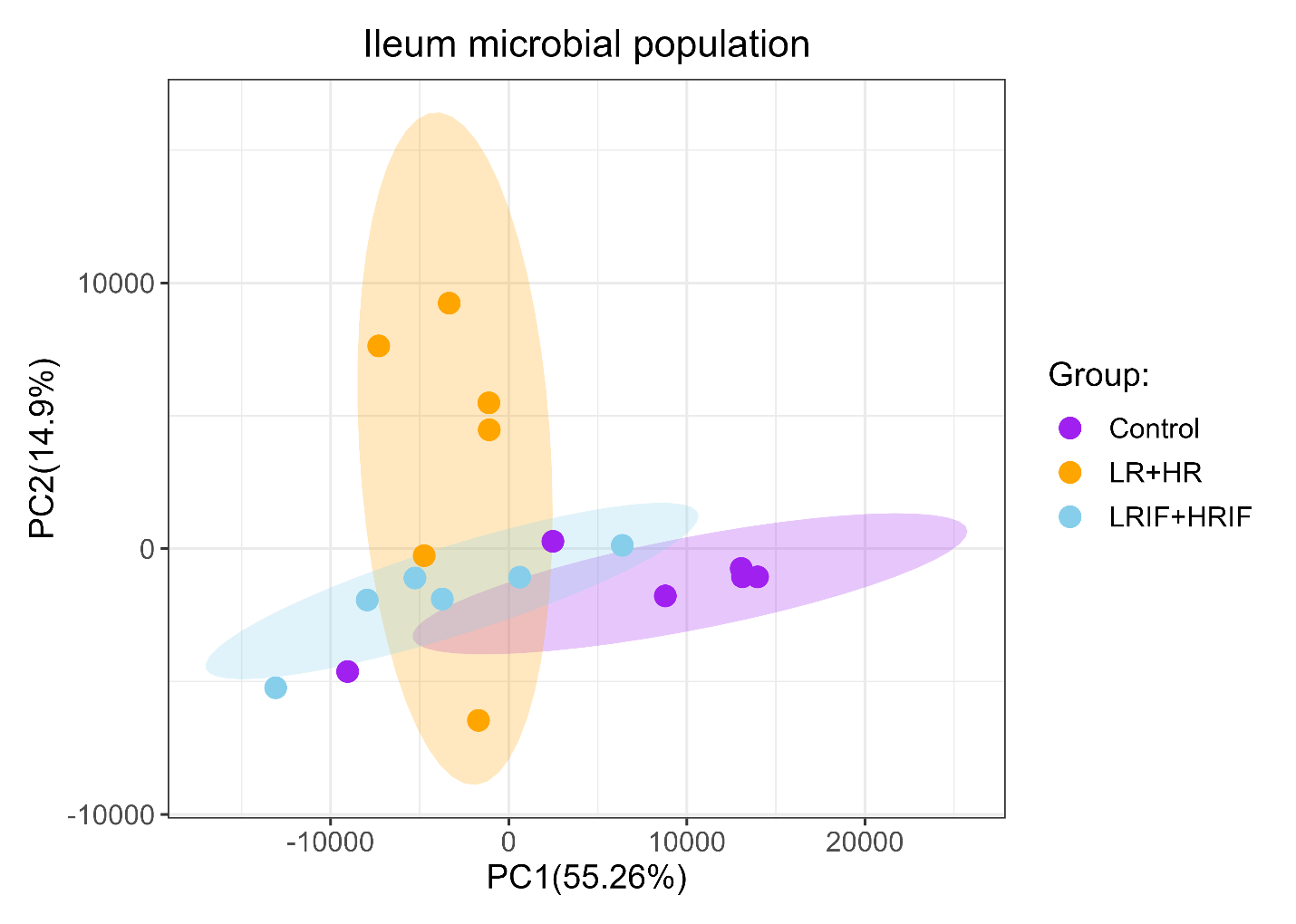


**Supplement Figure 2.** Beta-diversity of representative bacterial was assessed through conducting the PCA based on Bray-Curtis dissimilarity. The representative samples from microbial community among the LR+HR, LRIF+HRIF, and control groups were different in ileum.


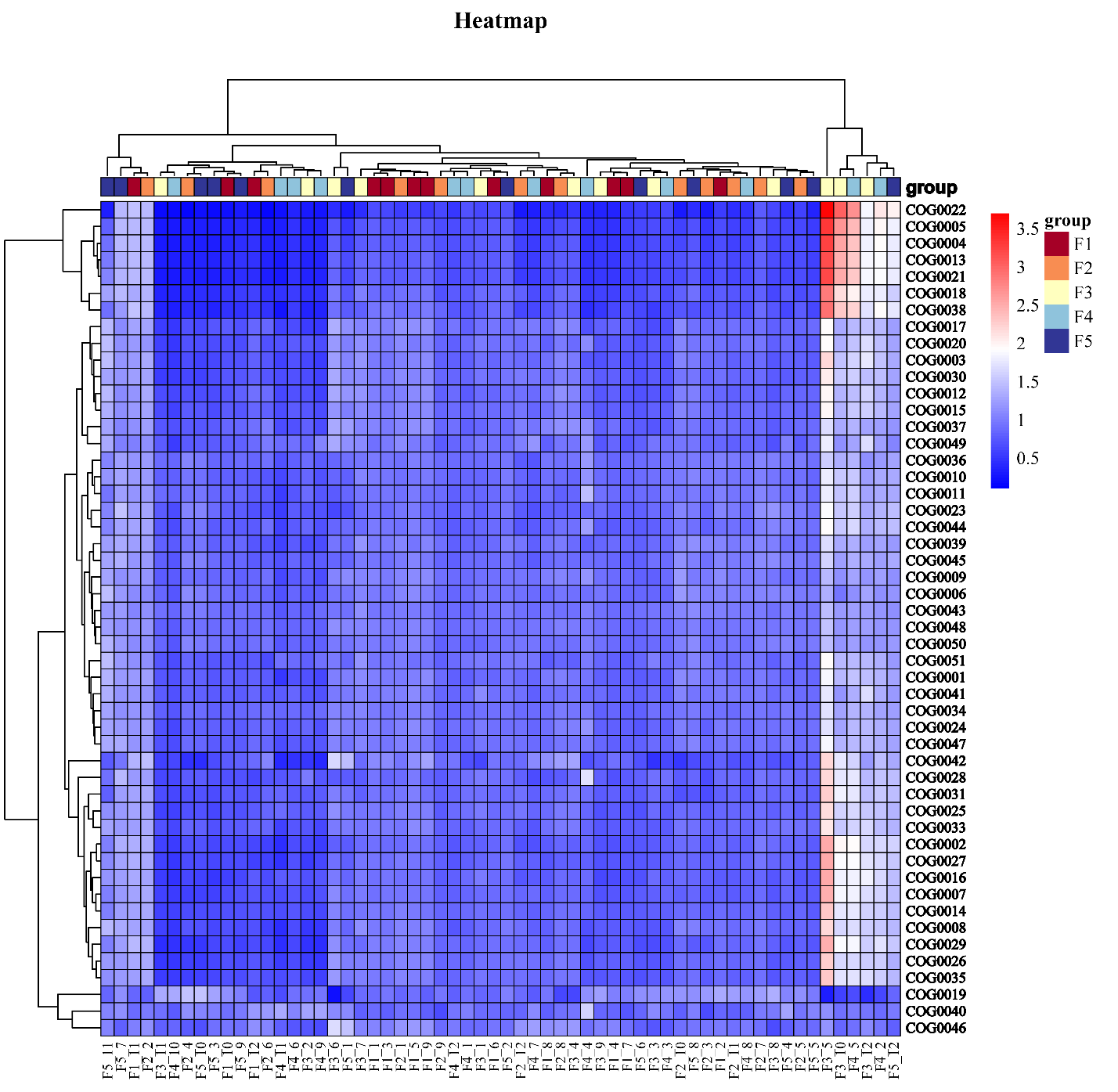


**Supplement Figure 3.** The top 50 bacterial functional descriptions annotated by PICRUSt in feces. (The predictions have 95% probabilities in truly reflecting the real functions of a gut microbiota (p < 0.05)).


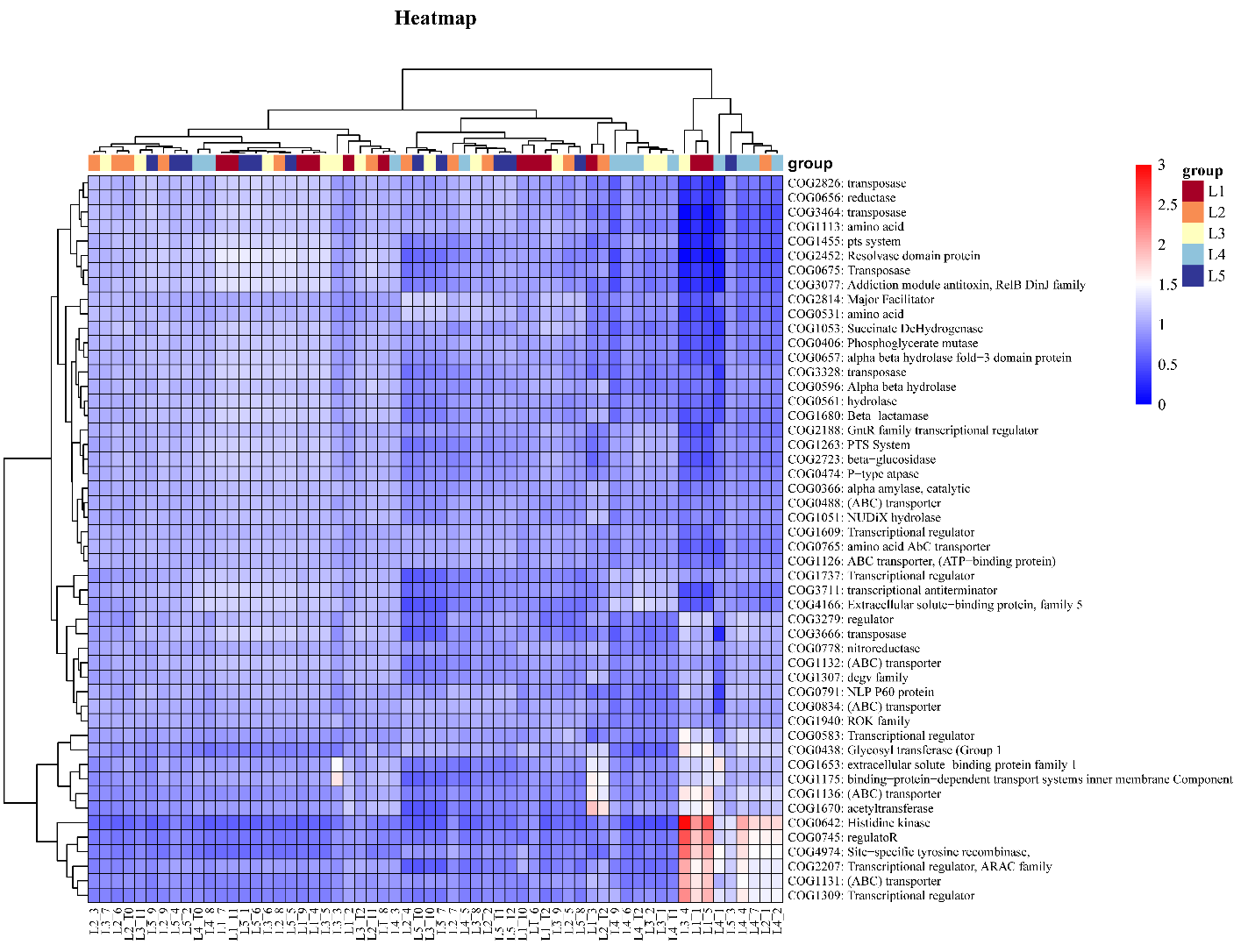


**Supplement Figure 4.** The top 50 Bacterial functional descriptions for the core microbiota annotated by PICRUSt in ileum. (The predictions have 95% probabilities in truly reflecting the real functions of a gut microbiota (p < 0.05)).


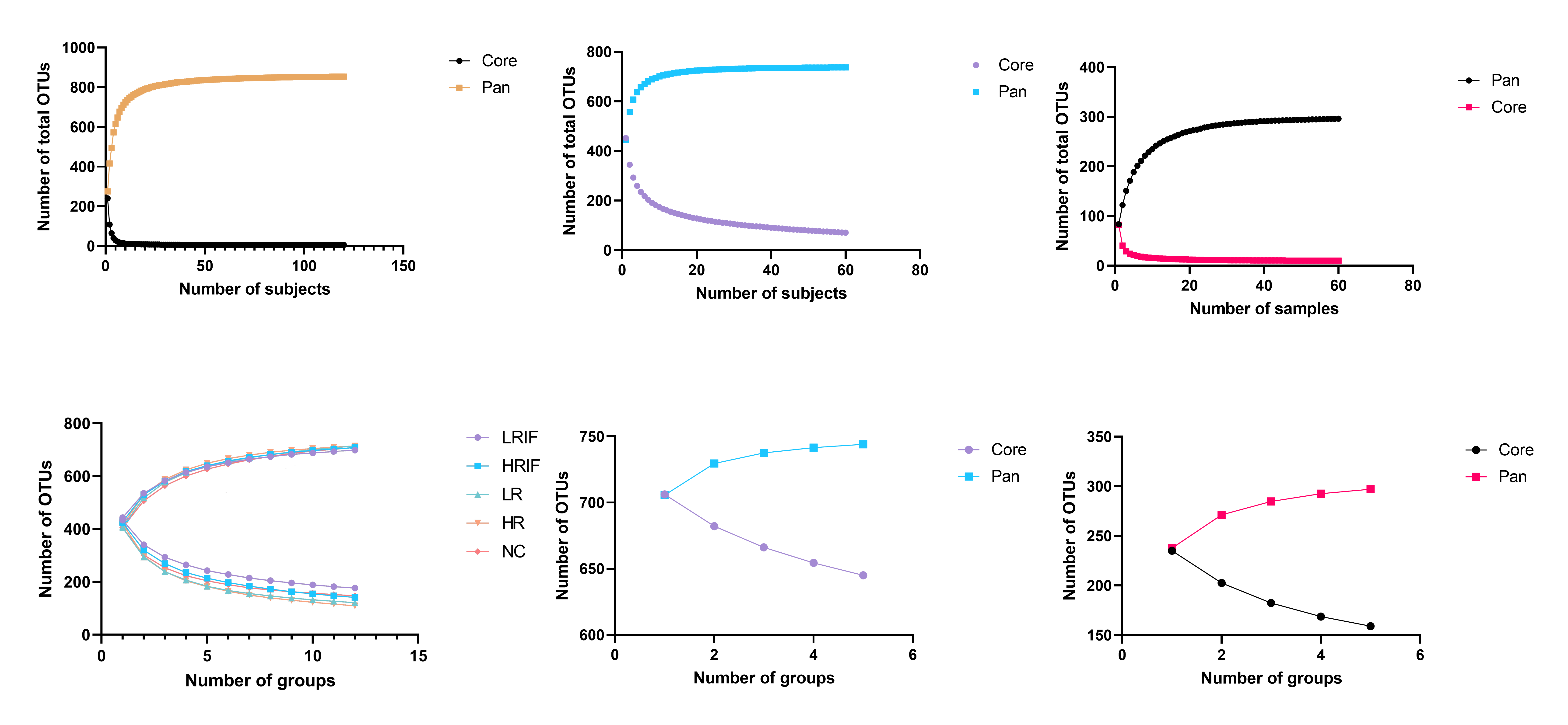


**Supplement Figure 5.** Pan and core OTUs of fecal and ileal samples. A. Pan and core OTUs of all samples; B. Pan and core OTUs of fecal samples; D. Pan and core OTUs of ileal samples; E. Pan and core OTUs of five groups; F. Pan and core OTUs of fecal samples in five groups; G. Pan and core OTUs of ileal samples in five groups.
